# No added value for Computer-Assisted surgery to improve femoral component positioning and Patient Reported Outcomes in Hip Resurfacing Arthroplasty; a multi-center randomized controlled trial

**DOI:** 10.1186/s12891-019-2883-7

**Published:** 2019-10-25

**Authors:** M. C. Koper, M. Reijman, E. M. van Es, J. H. Waarsing, H. W. J. Koot, S. B. Keizer, I. Jansen, F. C. van Biezen, J. A. N. Verhaar, P. K. Bos

**Affiliations:** 1 Department of Orthopedics, Erasmus University Medical Center, PO BOX 2040, 3000 CA Rotterdam, The Netherlands; 2 Department of Orthopedics, Maxima Medical Center, Eindhoven, The Netherlands; 3 Department of Orthopedics, Medical Center Haaglanden, Den Haag, The Netherlands; 4 Department of Orthopedics, Admiraal de Ruyter Hospital, Goes, The Netherlands

## Abstract

**Background:**

Computer Assisted Surgery (CAS) has proven to improve the accuracy in several orthopedic procedures. Therefore we used this technique to evaluate femoral component positioning in Hip Resurfacing Arthroplasty (HRA). The aim of this study was to evaluate imageless CAS compared to manually implanted femoral components and subsequently evaluates Patient Related Outcome Measures (PROMs). We hypothesized that the use of CAS optimizes the position of the femoral component and improves PROMs.

**Methods:**

This is a multicenter, single-blinded, randomized, controlled trial of two groups. In the CAS group guiding of the femoral component was done with imageless navigation. In the Conventional (control) group the femoral component was placed manually according to the preplanned position. The primary outcome measure consists of a maximum of 3 degrees difference between the postoperative Stem Shaft Angle (SSA) and preplanned SSA. Secondary outcome measures consist of the Hip disability and Osteoarthritis Outcome Scale (HOOS), the Harris Hip Score (HHS) and Visual Analogue Scale (VAS) pain score.

**Results:**

A total of 122 patients were randomized, 61 in the CAS group and 61 in the conventional group. There was no significant differences in accuracy of femoral implant position. The mean difference between the postoperative- and preplanned SSA was − 2.26 and − 1.75 degrees (more varus) respectively in the CAS and Conventional group. After surgery both groups show significant improvement in all PROMs compared to the baseline measurements, with no significant differences between the groups.

**Conclusion:**

Our cohort indicates no benefit for the use of CAS in accuracy of placement of the femoral component in HRA compared to manual implantation. There are no clinical differences in PROMs after 1 year follow up. This study showed no added value and no justification for the use of CAS in femoral component positioning in HRA.

**Trial registration:**

This trial is registered at ClinicalTrails.gov (https://clinicaltrials.gov/) on the 25th of October 2006: NCT00391937.

**Level of incidence:**

Level IIb, multicenter randomized controlled trial.

## Background

Hip Resurfacing Arthroplasty (HRA) is still considered a viable treatment option for young and active patients with end-stage osteoarthritis of the hip. Initially, this Metal-on-Metal (MoM) articulation showed promising short-term results, with high early return to work rates and high rates of participation in sports activities [1–3]. However, there have been a high number of early failures and a high revision rates [4–8]. This led to a recall of several MoM hip bearings, a more frequent follow-up of patients, and finally to a reduced use of HRA’s worldwide. Nevertheless, several HRA’s, are still used and reasonable survival rates have been reported. For some implants and patient categories equal to Total Hip Arthroplasty [9, 10].

The implantation of a HRA is a challenging procedure, due to reduced visibility and little exposure of the hip joint. A non-optimal placement of the femoral component is related to early femoral neck fractures, loosening, notching and higher risk of impingement with increased wear [11–14]. Therefore, an optimization of positioning of the femoral component in HRA could increase the survival of this bearing and possibly improve Patient Reported Outcomes (PROs).

Computer-Assisted-Surgery (CAS) was introduced to improve the accuracy of component positioning and survival of orthopedic implants. CAS has shown to result in an optimization of implant positioning in total hip arthroplasty [15–17] and an accurate component positioning in HRA’s [18–22]. However, there is no clear evidence that CAS improves the femoral positioning in HRA compared to manual placement.

Therefore, in this multi-center, patient-blinded, randomized controlled trail (RCT) we compared femoral component positioning between CAS and manual placement. The primary outcome measure was ability to achieve a postoperative Stem-Shaft Angle (SSA) within 3 degrees of the preplanned SSA. Secondly, we compared different PROMs between the two groups. We hypothesized that CAS results in a more accurate femoral component position and improves PROMs within one-year follow-up.

## Methods

### Study design

All consecutive patients who received an Articular Surface Replacement (ASR) prosthesis (DePuy International Ltd., Leeds, UK) were recruited between October 2006 and January 2010. Patients under the age of 60 (men) and 55 (women) years with nocturnal pain and/or limited walking distance, osteoarthritis (Kellgren Lawrence grade ≥ 2) of the hip, resistant to conservative treatment and eligible for a resurfacing hip prosthesis were asked to participate. Exclusion criteria consisted of a contralateral total hip prosthesis, body mass index > 30 kg/m^2^, request to correct an existing leg length discrepancy, not willing to participate in follow-up, proven metal allergy, evident osteoporosis, pathology of the acetabulum (evident acetabular dysplasia: CE angle of < 15 degrees, hip dysplasia, slipped capital femoral epiphysis and Legg-Calve-Perthes disease), previous hip surgery, vascular deficiency of the lower extremity, renal deficiency, active local or systemic infection, use of steroids and/or immunosuppression, femoral anatomic anomaly, femoral head neck ratio < 1, and extreme varus position (neck-shaft angle < 110 degrees). Conservative treated acetabular fractures were not excluded.

Patients were randomized using concealed allocation via a specifically designed website. Stratification took place per orthopaedic surgeon. All patients were blinded for the allocation, whereas the surgeon could not be blinded for the procedure. A standardized anteroposterior (AP) pelvic X-ray was used for calculation of the Centrum-Collum-Diaphysis (CCD)-angle and for preplanning of the femoral component. The software used for the preplanning was OrthoView (OrthoView, Meridian Technique Limited, Southampton, United Kingdom). Power analysis calculated a minimal of 117 patients per group in order to show a mean absolute difference of minimally 3 degrees between the postoperative SSA and preplanned SSA (one-side testing alpha = 0.05 and beta = 0.80). This sample size calculation is based on the study of Beaule et al., were they investigated the relation between the orientation of the femoral component and outcome of an ASR prosthesis [12]. With a follow-up period of three years, a 20% dropout was calculated and an inclusion of a total of 280 patients (140 each group) needed.

### Surgical planning and technique

Eleven experienced orthopedic hip replacement surgeons were trained to use the CAS-system. They attended an obligatory hands-on instructional cadaver course and a saw bone training. All operations were performed using a standard posterolateral approach. In the CAS group, surgical guiding of the femoral component was done with BrainLab Ci™ ASR System 1.0 (BrainLAB AG, Feldkirchen, Germany). There was no additional dissection necessary for CAS compared to the standard hip resurfacing surgery. Both groups received identical antibiotic prophylaxis with Cephalosporin (1000 mg) direct preoperatively and 24-h postoperatively. Thrombosis prophylaxis with Nadroparine was given until 6 weeks postoperatively. A standardized pain medication protocol was used postoperatively. Patients were rehabilitated under the guidance of the physiotherapist with immediate unrestricted weight bearing.

### Radiological evaluation

**Fig. 1:**
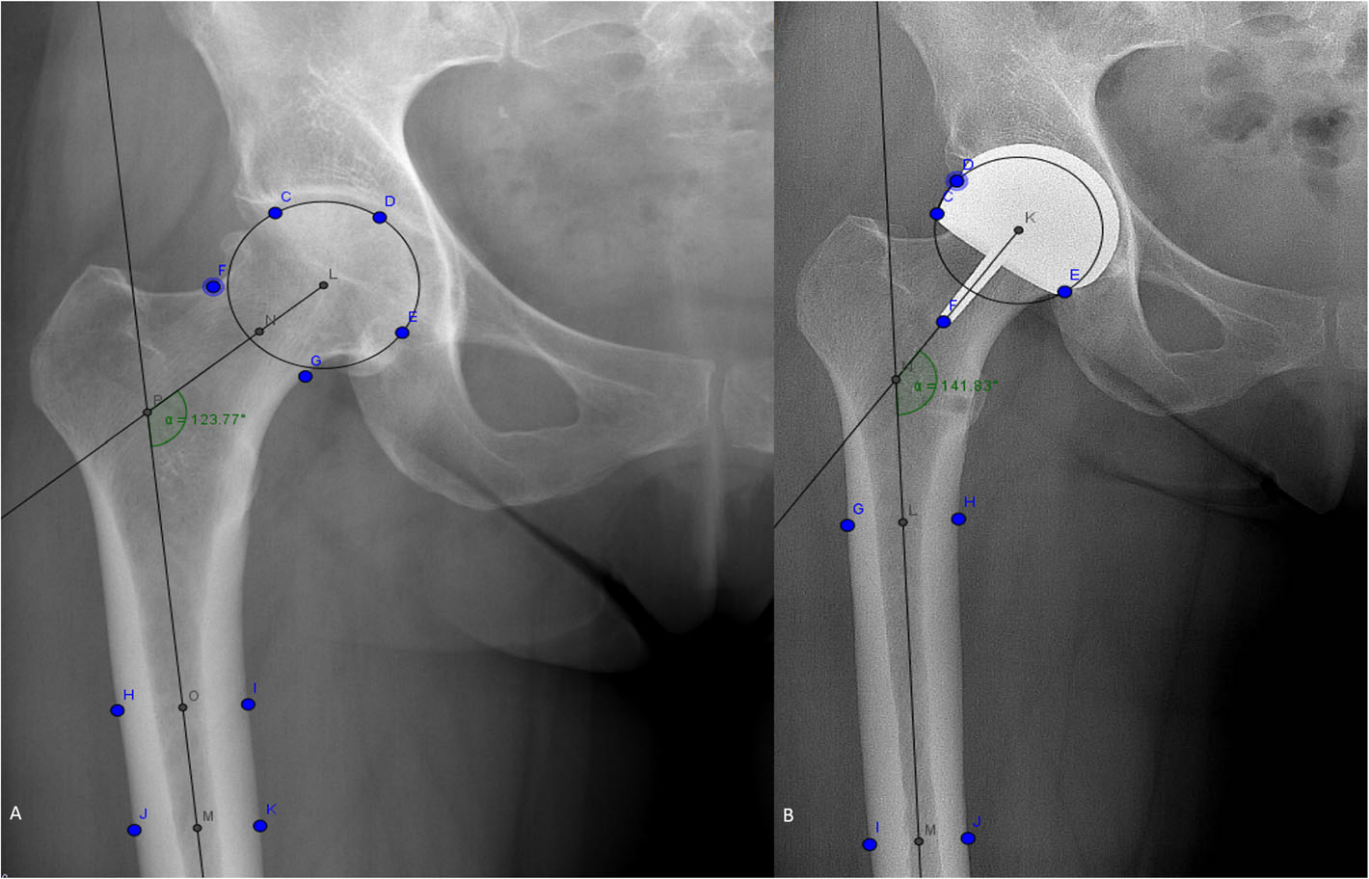
Examples of the use of GeoGebra (International GeoGebra Institute and GeoGebra GmbH, freeware) to calculate the Center-Collum-Diaphysis (**a**) and the postoperative Stem-Shaft-Angle (**b**)

To calculate the CCD-angle, the preoperative standardized AP-pelvic X-ray was analyzed in a blinded manner by two of the authors (MCK, EvE) using GeoGebra (International GeoGebra Institute and GeoGebra GmbH, freeware). Figure 1a demonstrates the use of GeoGebra where multiple marks are placed on the collum and the shaft to calculate the CCD angle. The SSA, defined as the angle between the stem of the femoral HR component and the axis of the femoral diaphysis in the AP projection, was measured on the preplanned AP-pelvic X-ray and direct postoperative AP-pelvic X-ray (Fig. 1b).

### Clinical evaluation

The Hip disability and Osteoarthritis Outcome Scale (HOOS), the Visual-Analogue-Scale (VAS) pain score and the Harris-Hip-Score (HHS) were used to evaluate relevant patient-centered outcomes.

The HOOS is subcategorized in five domains; pain, symptoms, function in daily life, sports and hip related quality of life. Scores on the HOOS range from 0 to 100, where 0 indicates the worst possible outcome and 100 the best possible [23]. The VAS pain is a validated tool to evaluate pain perception of a patient, and scores range from 0 to 10, with 0 indicating no pain an 10 being the worst pain experienced [24]. At each outpatient visit the HHS was completed by the orthopedic surgeon and used to score the hip function [25]. The survey has 10 questions and score a range from 0 to 100 with higher scores represent less dysfunction and better outcome.

### Data collection

Surgical blood loss and surgery duration were logged by the anesthesiologist and written on the surgery evaluation form. Each adverse event was classified as ‘surgical’ when it occurred in the operation room, as ‘early’ when it occurred within three months after surgery, and as ‘late’ when it occurred more than three months postoperatively. At the end of the trial, all hospital records of the participating patients were retrieved and checked to verify the adverse events and their extensiveness.

Baseline questionnaires were administered before surgery, and subsequently at 6 weeks, 3 and 12 months postoperative. At each outpatient visit, the HHS was completed by the orthopedic surgeon. The other questionnaires were patient-reported and were sent out electronically (web-based or via email) or sent on paper by post.

### Statistical analysis

Descriptive statistics including means, standard deviations, frequencies and percentages were used to describe the patient characteristics. For all X-ray measurements the intra-observer and inter-observer reliability were evaluated using the intra-class correlation coefficient (ICC). We used a two-way mixed model with absolute agreement and a confidence interval of 95%. The ICC values range from 0 to 1, in which 1 indicates perfect reliability and an ICC greater than 0.75 considered acceptable [26].

Intention-to-treat analyses were used for all variables. However, due to some protocol violations, all data were also analyzed per protocol. The independent t-test was used to assess differences between groups for continuous data, while the Chi-square test was used to assess differences in categorical data. To assess differences in continuous data over time within the same treatment group, a paired t-test was used. For the implant survival analysis, a Kaplan-Meier was used to compare treatment groups. Events were defined as revisions of the femoral and/or acetabular component for any reason, and patients without an event were censored at 3 year postoperative. All analyses were performed using SPSS 20(IBM Corporation, Armonk, NY). All tests were two-sided and a *p*-value < 0.05 was considered significant.

## Results

**Fig. 2:**
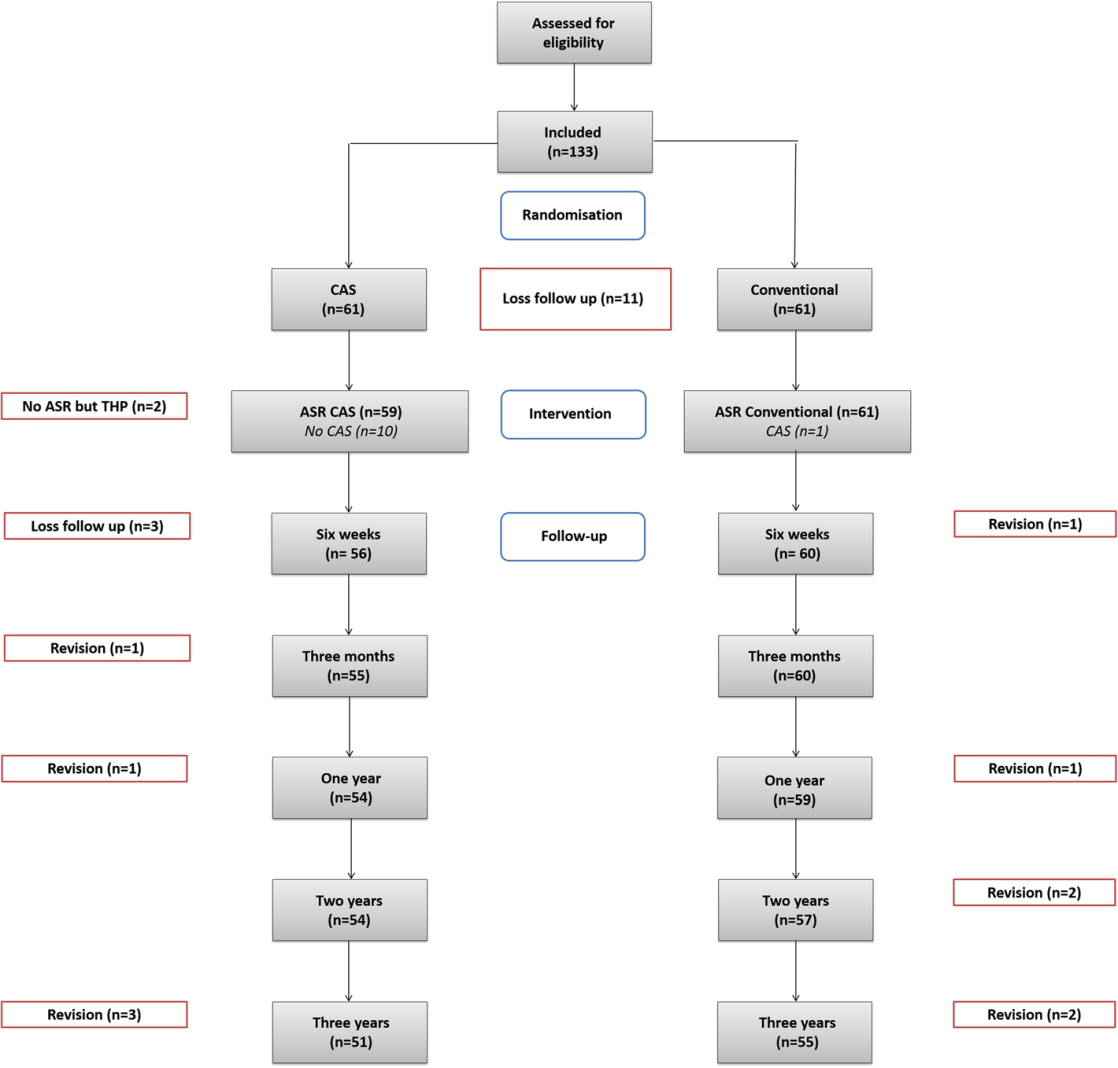
Study flowchart

During the trial period, a total of 125 patients (133 hips) were included, 67 hips were randomized to the conventional group and 66 hips to the CAS group. The study flowchart is depicted in Fig. 2 and patient characteristics in Table 1. A total of 11 randomized patients were excluded due to primary missing data and loss of follow up, five patients in the conventional group and six patients in the CAS group. These patients showed no difference in baseline characteristics. In general, patients in both groups were similar, except for BMI, which was significantly higher in the CAS group (26.9 versus 25.5, *p* = 0.003), which can be explained by a higher body weight (Table 1). Unfortunately, due to an international recall of the ASR prosthesis after publications of high complication and failure rates the study was prematurely ended. This resulted in a lower number of inclusions needed and incompleteness of data gathered by the participated orthopedic surgeons.

**Table 1:** Baseline patient characteristics for the CAS and Conventional group

	CAS (*n* = 61)	Conventional (*n* = 61)	*P*-value	Excluded hips (*n* = 11)
Age (years) (SD: range)	50 (6.3: 22 to 60)	50 (6.4: 29 to 60)	0.887	45.64 (6.9: 37–59)
Weight (kg) (SD: range)	85.6 (11.3: 62–107)	79.7 (12.27: 53–110)	0.006*	77.9 (11.5: 55–95)
Length (cm) (SD: range)	178.3 (8.9: 161–196)	176.2 (9.2: 157–196)	0.210	175.2 (10.5: 164–197)
BMI (kg/m^2^) (SD: range)	26.9 (2.6: 20.3 to 30.1)	25.5 (2.4: 20.4–29.4)	0.003*	25.3 (2.9:19.0–29.8)
Gender (Men: Women)	39: 22	42: 19	0.702	6:5
Side (L: R)	25:36	29:32	0.585	7:3

*CAS* Computer-Assisted-Surgery, *BMI* Body Mass Index

Age, Weight, Length and BMI are presented as means. Gender and Side are given as a ratio

### Surgical details

Table 2 shows the details on the surgical procedure for each group. The mean operation time in the CAS group was significantly(*p* < 0.001) longer, i.e. 19 min. Three minor ‘early’ adverse events were reported, all in the conventional group. One patient had minor cardiac ischemia, the second patient complained of a painful lower leg and swelling, but thrombosis was excluded. The third patient had a superficial skin infection and required oral antibiotics. All resolved without further problems.

**Table 2:** Surgery details of the CAS and Conventional groups

	CAS (n = 61)	Conventional (*n* = 61)	*P-*value
Surgery time (min) (SD: range)	116 (30: 65–240)	97 (24: 60–180)	0.000*
Blood loss (mL) (SD: range)	645 (276: 200–1500)	573 (282: 150–1500)	0.171
Component size (mm) (SD: range)	49 (3: 43–57)	49 (3: 41–57)	0.635
CAS protocol deviations	12	1	
- Conventional /CAS	10	1	
- Total Hip Prosthesis	2	0	

*CAS* Computer-Assisted-Surgery

Surgery time, Blood loss and Component size are given as means. CAS protocol deviations are given as counts

* Significant difference between CAS and Conventional group, *P* < 0.000

Protocol violations occurred thirteen times. Ten of the CAS randomized patients were operated without CAS due to no CAS system availability during surgery. Two patients in the CAS group were excluded because safe femoral component placement was considered not possible and a total hip prosthesis was implanted. One conventional randomized patient was operated with CAS.

### Radiographic evaluation

The intra-observer reliability for the two readers was excellent: 0.98, 95% CI 0.94–0.996 for reader 1, and 0.96, 95% CI 0.91–0.99 for reader 2. The ICC for the inter-observer reliability was 0.95, 95% CI 0.89–0.99. The mean native CCD-angle was 129 degrees in both groups, with no significant difference between the groups. We did find a significant difference (*P* = 0.033) in the preplanned SSA within the intention to treat analysis. This is a baseline difference and we do not have a clear explanation for this and believe this is not of any clinical relevance for the outcome of this study. The mean postoperative SSA minus the preplanned SSA showed no significant difference between the two groups (*p* = 0.636). A slightly more varus position was found in both groups with − 2.26 and − 1.75 degrees deviation respectively in the CAS and conventional group. Analysis of patients with more than 3 degrees, 7 degrees or 10 degrees deviation also showed no significant difference. Table 3 shows all measured data calculated as intention to treat, as well as calculations per protocol.

**Table 3:** Radiographic evaluation of the angles

	Radiographic evaluation angles (shown as intention to treat)			Radiographic evaluation angles (shown per protocol)		
	CAS (n = 61)	Conventional (n = 61)	*P* Value	CAS (*n* = 50)	Conventional (*n* = 70)	*P* Value
CCD-Angle, degrees (SD: range)	129,5 (6.1: 117–143)	128,6 (6.5: 115–149)	0.443	129,2 (6.1: 117–143)	128,9 (6.5: 115–149)	0.780
Preplanned SSA, degrees (SD: range)	138,3 (3.8: 128–148)	136,6 (4,8: 127–152)	0.033*	138,0 (3.8: 128–148)	137,1 (4,7: 127–152)	0.281
Post-operative SSA, degrees (SD: range)	136,0 (5.7: 124–150)	134,9 (6,7: 119–153)	0.311	136,3 (5.6: 124–150)	134,8 (6,6: 119–153)	0.196
Difference postoperative SSA minus preplanned SSA						
- Mean, degrees (SD: range)	−2.26 (5.8: −15.25 –12.11)	−1.75 (5.9: − 13.14 –16.21)	0.636	− 1.7 (5.9: − 15.25 – 12.11)	− 2.2 (5.8: − 13.14 – 16.21)	0.592
- Absolute, degrees (SD: range)	5.14 (3.5: 0.07–15.25)	4.94 (3.5: 0.04–16.21)	0.768	5.0 (3.5)	5.0 (3.5)	0.932
- > 3 degrees, n (%)	44 (72%)	40 (66%)	0.558	35 (70%)	44 (61%)	0.692
- > 7 degrees, n (%)	18 (29%)	19 (31%)	0.884	12 (24%)	20 (28%)	0.534
- > 10 degrees, n (%)	6 (10%)	4 (7%)	0.517	05 (10%)	10 (14%)	0.586

CAS = Computer-Assisted-Surgery, CCD = Centrum-Collum-Diaphysis, SSA = Stem-Shaft-Angle.* significant difference

### Clinical evaluation

Compliance rates for the different questionnaires ranged between 87 and 100% at baseline, 70–90% after 6 weeks, 70–90% after 3 months and 67–90% after 12 months of follow-up. Reasons for missing data are the international recall of the prosthesis and shutdown of the study website. Table 4 describes all results of the questionnaires during the one year follow-up visits, separately for the two groups.

**Table 4:** Patient Reported Outcomes with one year follow up. Calculated per protocol

		Baseline		Six weeks		Three months		One year		*P*-value (LMM)
		CAS	Conventional	CAS	Conventional	CAS	Conventional	CAS	Conventional	
VAS		5.7 (1.9)	5.4 (2.0)	1.3 (2.0)	1.3 (1.8)	0.8 (1.3)	0.8 (1.3)	0.4 (1.0)	0.5 (1.2)	0.688
HOOS	Pain	38.4 (13.0)	40.5 (15.4)	81.1 (15.5)	79.2 (13.3)	87.0 (15.6)	86.5 (14.7)	91.1 (11.2)	88.0 (16.7)	0.432
	Other symptoms	35.0 (14.4)	35.7 (14.2)	67.2 (16.7)	69.0 (15.5)	72.5 (16.1)	72.2 (16.1)	74.5 (18.0)	75.9 (19.6)	0.914
	Activities of daily living	38.2 (14.9) *	42.7 (16.4) *	72.8 (17.3)	71.2 (13.5)	83.2 (17.6)	82.2 (15.2)	89.6 (11.7)	87.3 (16.3)	0.333
	Sport	16.6 (14.0) **	22.8 (18.3) **	53.6 (29.2)	46.2 (25.6)	69.9 (26.0)	65.8 (25.6)	73.8 (24.2)	76.6 (23.2)	0.444
	Hip-related QoL	21.7 (12.3)	24.5 (11.7)	51.9 (16.1)	48.4 (16.7)	66.5 (20.2)	59.6 (17.9)	71.9 (14.6)	69.0 (20.9)	0.309
HHS	Total	57.1 (10.6)	60.6 (10.6)	79.1 (16.6)	80.2 (11.5)	91.0 (12.8)	93.7 (8.7)	96.3 (7.1)	97.8 (4.0)	0.537

*CAS* Computer-Assisted-Surgery, *LMM* linear mixed-model, *VAS* visual analogue scale. HOOS=Hip disability and Osteoarthritis Outcome Scale (HOOS). HHS=Harris Hip Score. All data are presented as means (SD). * significant difference (*p* = 0.028). ** significant difference (*p* = 0.021)

The baseline mean VAS score in both groups decreased significantly(*P* = 0.000) after 6 weeks of surgery. Between both groups no significant difference at any time point was observed. The HOOS questionnaire at baseline showed no differences between the CAS and conventional group in pain, hip-related quality of life and other symptoms. The conventional group showed significant higher scores in the subscales activities of daily living(*P* = 0.028) and sport(*P* = 0.021) at baseline. After 6 weeks, 3 months and one year follow up, no significant differences between the two groups were observed.

The mean HHS was significantly increased in both groups after six weeks(*P* = 0.000), three months(*P* = 0.000) and 1 year(*P* = 0.026) of surgery.

### Survival analysis

**Fig. 3:**
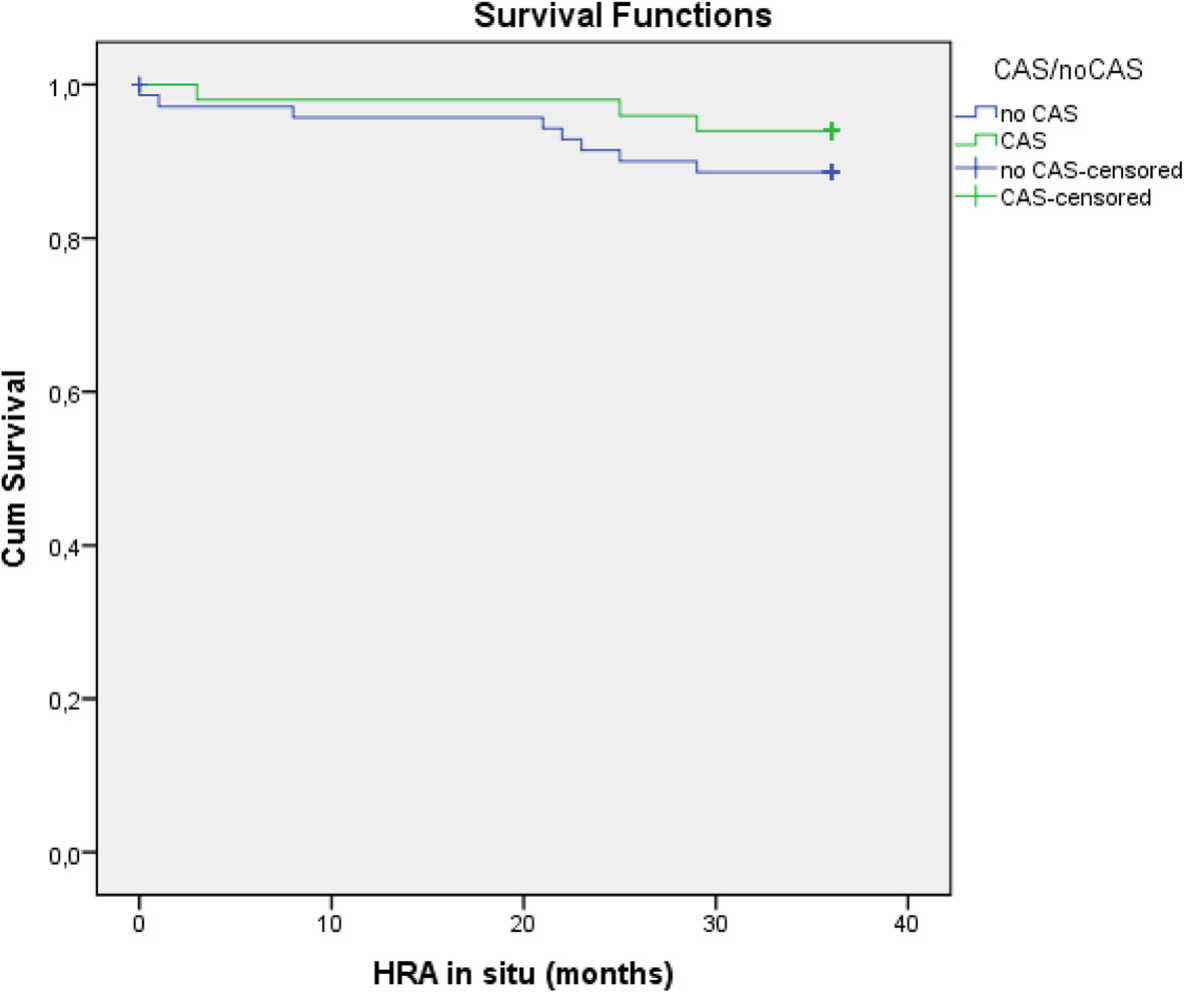
The 3 year survival Kaplan-Meier curve between the CAS and Conventional group. No significant difference (*p* = 0.304) in survival was found

During a three-year follow-up period, 11 revisions were performed. An overall survival of 91% in three years was calculated in the entire group. Table 5 shows the revision characteristic between the two groups. All late events in our clinics were managed with a conventional total hip arthroplasty. With per protocol analysis we found more revisions in the conventional group versus the CAS group (8 versus 3) in the three-year follow-up period, this difference was not significant. Figure 3 shows the Kaplan-Meier survival curve between the two groups.

**Table 5:** Revision characteristics for the CAS and Conventional group

	Age/Gender	Size component (mm)	Revision indication	Time to revision (months)	Component revised	Anatomy (degrees)	Angle planned (degrees)	Angle post (degrees)
CAS randomized	46/Female	46	Collum fracture	3	Femur	Normal (128)	133	124
	56/Male	49	Collum fracture	25	Femur	Coxa Valga (136)	142	135
	51/Male	53	ARMD, high cobalt/chromium	29	Femur + Acetabulum	Normal (130)	136	142
	54/Female (no CAS)	47	Aseptic loosening	30	Femur + Acetabulum	Normal (129)	134	131
	53/Female (no CAS)	46	Collum fracture	1,5	Femur	Coxa Valga (141)	143	**–**
Conventional randomized	52/Male	49	Pain	25	Femur	Normal (122)	137	133
	55/Female	45	Aseptic loosening	8	Femur + acetabulum	Normal (127)	133	141
	53/Female	43	Aseptic loosening	21	Femur + Acetabulum	Normal (126)	136	140
	59/Male	51	Pain, high cobalt/chromium	23	Femur + Acetabulu	Normal (127)	138	136
	54/Female	41	ALTR	22	Femur + Acetabulum	Coxa Valga (135)	132	149
	48/Male	51	Aseptic loosening	0.5	Acetabulum	Normal (134)	138	134

*CAS*, Computer-Assisted-Surgery, *ARMD* Adverse Reaction to Metal Debris

## Discussion

In this multi-center, patient-blinded, randomized controlled study we compared imageless CAS versus manual placement of the femoral component in HRA. The primary endpoint of this study was an accurate placement of the femoral component within 3 degrees difference between the postoperative SSA and preplanned SSA. We did not find a difference in accuracy between the CAS and conventional group.

An accurate positioning of the femoral component in HRAs remains a critical step during surgery. A non-optimal placement of the femoral component is related to early failure. An excessive valgus position results is an increased risk of femoral notching and weakening of the bone with possible avascular necrosis, while a varus position leads to increased femoral neck fractures and aseptic loosening [11, 13, 14, 27, 28]. Increased metal ion levels, adverse reaction to metal debris (ARMD) and pseudotumor formation also seem related to a suboptimal position of components, which may result in increased revision rates [5, 29, 30]. The importance of CAS in component placement in HRA is already shown in preclinical and clinical studies [18, 31–36]. However, most of these clinical studies retrospectively evaluated case series. In this RCT the CCD-angle, preplanned SSA and postoperative SSA were all determined with high intra- and interobserver reproducibility, showing the accuracy of our measurements. The CCD angle in our study was similar for the two treatment groups. We only found a small but significant difference in the preplanned SSA (*P* = 0.003) between the two groups; 138 degrees in the CAS group compared to 137 degrees in the conventional group. However, we consider this difference not of clinical significance. We did not observe any difference in the mean postoperative SSA between the two treatment groups, nor in the number of hips with a postoperative difference in SSA of ≥3, ≥7 or ≥ 10 degrees from the preplanned SSA. These results show that CAS did not result in an increased accuracy in placement of the femoral component. In contrast to our results, Stiehler et al., did show a significant improvement in placement of the femoral component with the use of CAS. Fewer femoral components were positioned in ≥5 degrees absolute deviation compared to preplanning in the CAS group [19]. In another, retrospective study, they showed a more accurate placement of the femoral component and less deviations from the planned SSA was accomplished with the use of CAS [37].

The impact of CAS on several aspects of patients’ functioning (HHS, HOOS and VAS) was evaluated during a one-year follow-up period. Although the patients differed in their level of activities of daily living and sport at baseline, these differences were not clinically relevant. We did observe an overall improvement of patients’ functioning over time, but this was similar for the two treatment groups. All results are consistent with previous studies [9, 19, 37].

Our study has several limitations. Unfortunately due to recall of the ASR system, the study was prematurely terminated, resulting in a lower number of patients than needed, possibly hampering our statistical analysis. Selective protocol deviations due to incidentally unavailability of the CAS system in certain surgeries possibly influenced our study outcome. In this case, per protocol analysis would provide a better estimate of the effects of this method. Lastly, our longitudinal analysis of PROs was hampered by missing data. As missing data occurred due to termination of the study, selective bias will be limited, as patients who completed the data are representative of the study population.

## Conclusions

Despite the limitations and recall of the ASR prosthesis we feel obligated to present our results. As orthopedic surgeons we have to strive to perform better and always search for optimization of a procedure. In our study, we show no added value for the use of imageless CAS in placement of the femoral component. In addition, CAS also did not improve any of the Patient-Centered Outcomes after one year follow up. Therefore we do not expect that CAS will result in long-term event-free survival, but this remains to be determined in long-term follow up.

## Data Availability

All data are stored at different secured servers in the Erasmus University Medical Center to ensure the safety and de-identification. To access the data a written request can be send to the Erasmus University Medical Center, Department of Orthopedics, PO Box 2040, 3000 CA Rotterdam, the Netherlands.

## Abbreviations

AP: Anteroposterior
ARMD: Adverse Reaction to Metal Debris
ASR: Articular Surface Replacement
BMI: Body Mass Index
CAS: Computer Assisted Surgery
CCD: Centrum-Collum-Diaphysis
HHS: Harris Hip Score
HOOS: Hip disability and Osteoarthritis Outcome Scale
HRA: Hip Resurfacing Arthroplasty
ICC: Intra-Class Correlation
MoM: Metal-on-Metal
PROMs: Patient Related Outcome Measures
PROs: Patient Reported Outcomes
RCT: Randomized Controlled Trial
SSA: Stem Shaft Angle
VAS: Visual Analogue Scale
